# Supplementary material for: Health inequities in influenza transmission and surveillance
Source: PLoS Comput Biol. 2021 Mar 11;17(3):e1008642. doi: 10.1371/journal.pcbi.1008642 (PMC7951825; doi:10.1371/journal.pcbi.1008642)
Supplement: S1 Appendix — Additional details of the implementation of the Exponential Random Graph Model (ERGM). (DOCX) [file pcbi.1008642.s041.docx]

**ERGM Model Details**

Due to the egocentric ERGM model, edge-based factors could not be included, therefore possession of home, school, or work contacts was considered a node level factor. An edge between two home nodes is assumed to be a home contact, an edge between two work nodes is assumed to be a work contact, an edge between two school nodes is assumed to be a school contact, and all other edges occur in other settings that are not explicitly considered in the network model. If individuals had any contacts reported at home, they were considered a "home" node. If any contacts were reported at school or work, they were considered either a "school" or "work" node, and individuals were assumed to attend only school or work. In some cases, contacts were reported in unexpected locations (i.e. infants/toddlers/children reporting work contacts or adults/elderly reporting school contacts) or contacts were reported in multiple locations (i.e. one individual reports contacts at both work and school with another individual). Though these situations may be possible, to simplify these reports, it was assumed that children's contacts occurred at school and adult's contacts occurred at work. Based on this data, we simulated ten unweighted, undirected networks with the same distributions of each of the previously mentioned factors as the POLYMOD data.
