# Supplementary material for: Health inequities in influenza transmission and surveillance
Source: PLoS Comput Biol. 2021 Mar 11;17(3):e1008642. doi: 10.1371/journal.pcbi.1008642 (PMC7951825; doi:10.1371/journal.pcbi.1008642)
Supplement: S2 Appendix — Additional details on the sensitivity analysis incorporating partial immunity into epidemiological simulations of influenza transmission on SES-heterogeneous networks. (DOCX) [file pcbi.1008642.s042.docx]

**Partial immunity Sensitivity Analysis Details**

We modeled 5 seasons of influenza strain circulation with a polarized partial immunity model, where each season, infected individuals become immune, and then lose immunity at rate η in each subsequent season cite (Bansal et al., 2010). Parameters were distributed based on SES on the ERGM-generated network as described above. We selected an immunity loss of 0.4 which assumes that 40% of infected individuals become fully susceptible by the subsequent influenza season (Barry et al., 2008). 20 simulations of 5 seasons were performed on each of 5 networks composed of 60% low SES population.

References

Bansal S, Pourbohloul B, Hupert N, Grenfell B, Meyers LA. The shifting demographic landscape of pandemic influenza. PLoS One 2010;5:1–8. https://doi.org/10.1371/journal.pone.0009360.

Barry JM, Viboud C, Simonsen L. Cross-Protection between Successive Waves of the 1918–1919 Influenza. J Infect Dis 2008;198:1427–34. https://doi.org/10.1086/592454.Cross-Protection.
