# Supplementary material for: Health inequities in influenza transmission and surveillance
Source: PLoS Comput Biol. 2021 Mar 11;17(3):e1008642. doi: 10.1371/journal.pcbi.1008642 (PMC7951825; doi:10.1371/journal.pcbi.1008642)
Supplement: S1 Table — Summary of ERGM model results, including each model factor, its coefficient estimate and standard deviation, its p-value and a brief interpretation. (DOCX) [file pcbi.1008642.s043.docx]

**ERGM model summary**

| Factor | Estimate | St. dev. | Interpretation | p-value |
| --- | --- | --- | --- | --- |
| Netsize.adj | 8.89 | 0 | 7290 nodes in the network. -ln(7290) = -8.89 | <0.0001 |
| Edges | 3.6 | 0.04 | Log odds of a tie occurring: 3.64. A dense network. | <0.0001 |
| Nodefactor: Female | 0.04 | 0.02 | A female node is 4 times more likely to appear in an edge in the network, compared to a male node. | <0.0001 |
| Nodefactor: Adult | 0.64 | 0.03 | An adult node is 0.64 times more likely to appear in an edge in the network, compared to infants and children. | <0.0001 |
| Nodefactor: Elderly | 0.48 | 0.04 | An elderly node is 0.48 times more likely to appear in an edge in the network, compared to infants and children. | <0.0001 |
| Nodefactor: Work | -0.81 | 0.03 | A work node is 0.18 times less likely to appear in an edge in the network, compared to a school node. | <0.0001 |
| Nodefactor: low education | -2.53 | 0.07 | A low education node is 2.53 times less likely to appear in an edge in the network, compared to a medium education node. | <0.0001 |
| Nodefactor: High education | -2.5 | 0.06 | A high education node is 2.5 times less likely to appear in an edge in the network compared to a medium education node. | <0.0001 |
| Nodematch: Age | 0.81 | 0.03 | An edge between nodes of the same age is 0.81 times more likely than nodes of different ages. | <0.0001 |
| Nodematch: Home | -2.0 | 0.02 | An edge between home nodes is 2 times less likely than between nodes in different or unknown locations. | <0.0001 |
| Nodematch: School/work | 1.98 | 0.04 | An edge between school nodes and work nodes is 1.98 times more likely than in other or unknown locations. | <0.0001 |
| Nodematch: Low education | 4.84 | 0.11 | An edge between low education nodes is 4.84 times more likely than between low education and a different or unknown education level. | <0.0001 |
| Nodematch: Medium education | -1.92 | 0.04 | An edge between medium educations nodes is 1.92 times less likely to occur than between a medium node and a node of a different or unknown education | <0.0001 |
| Nodematch: High education | 4.37 | 0.09 | An edge between high education node is 4.37 times more likely to occur than between a high education node and a node of a different or unknown education. | <0.0001 |
