# Supplementary material for: Health inequities in influenza transmission and surveillance
Source: PLoS Comput Biol. 2021 Mar 11;17(3):e1008642. doi: 10.1371/journal.pcbi.1008642 (PMC7951825; doi:10.1371/journal.pcbi.1008642)
Supplement: S2 Table — Higher values indicate greater correlation. VIF>20 is concerning. VIF >100 indicates severe multicollinearity. (DOCX) [file pcbi.1008642.s044.docx]

**Variance inflation factors for ERGM covariates**

| Model factor | VIF |
| --- | --- |
| Edges | 6.08 |
| Nodefactor: Female | 8.14 |
| Nodefactor: Adult | 2.33 |
| Nodefactor: Elderly | 15.83 |
| Nodefactor: Work | 6.13 |
| Nodefactor: Low education | 7.17 |
| Nodefactor: High education | 9.5 |
| Nodematch: Age | 5.44 |
| Nodematch: Home | 12.96 |
| Nodematch: School/work | 3.67 |
| Nodematch: Low education | 5.53 |
| Nodematch: Medium education | 4.57 |
| Nodematch: High education | 0 |
