# Supplementary material for: Health inequities in influenza transmission and surveillance
Source: PLoS Comput Biol. 2021 Mar 11;17(3):e1008642. doi: 10.1371/journal.pcbi.1008642 (PMC7951825; doi:10.1371/journal.pcbi.1008642)
Supplement: S3 Table — (DOCX) [file pcbi.1008642.s045.docx]

**Details of ERGM networks with added low SES nodes**

| Percent low SES | Number of nodes | Number of low SES nodes | Number of medium SES nodes | Number of high SES nodes | Number of NA SES nodes |
| --- | --- | --- | --- | --- | --- |
| Original model: ~10% | 7290 | 718 | 4170 | 1334 | 1068 |
| ~20% | 8726 | 2154 | 4170 | 1334 | 1068 |
| ~30% | 9444 | 2872 | 4170 | 1334 | 1068 |
| ~40% | 10880 | 4308 | 4170 | 1334 | 1068 |
| ~50% | 13752 | 7180 | 4170 | 1334 | 1068 |
| ~60% | 15906 | 9334 | 4170 | 1334 | 1068 |
