# Supplementary material for: Health inequities in influenza transmission and surveillance
Source: PLoS Comput Biol. 2021 Mar 11;17(3):e1008642. doi: 10.1371/journal.pcbi.1008642 (PMC7951825; doi:10.1371/journal.pcbi.1008642)
Supplement: S4 Table — Parameters are pertinent to influenza and to SES-based mechanisms. Parameters are defined as “high SES” and “low SES”, though some of the “high SES” parameters are found from literature describing the entire population, due to lack of value specifically pertaining to those of high SES. (DOCX) [file pcbi.1008642.s046.docx]

**Parameters for influenza network simulations**

| Parameter | Symbol | Value | Source | Effect |
| --- | --- | --- | --- | --- |
| High SES susceptibility | β_high_ | 0.04 | Derived from R0 and Network structure | Determines probability of transmission between infectious individual and susceptible, high SES neighbor |
| Low SES susceptibility | β_low_ | 0.08 | Cohen et al., 2008 | Determines the probability of susceptible lower SES individuals becoming infected by an infectious neighbor |
| Latent period | α | 1/0.5 * 7 | Carrat et al., 2008 | Determines rate at which exposed individuals become infected |
| High SES healthcare utilization infectious period | γ_high_ | 1/0.21 * 7 | Carrat et al. 2008 | Determines rate at which infected high SES individuals become recovered |
| Lowe SES healthcare utilization infectious period | γ_low_ | 1/0.14 * 7 | Pepin et al., 2013 | Determines the rate of low SES infectious individuals becoming recovered |
| High SES vaccination rate | δ_high_ | 0.21 | Linn et al., 2010 | Determines quantity of high SES nodes removed randomly from the network prior to epidemic simulation |
| Lower SES vaccination rate | δ_low_ | 0.33 | Linn et al. 2010 | Determines number of low SES nodes that are removed from the network prior to epidemic simulation |
| High SES sickness absenteeism rate | ρ_high_ | 0.74 | Piper et al., 2017 | Determines the rate that high SES infected individuals become absent and remove approximately 90% of edges |
| Low SES sickness absenteeism | ρ_low_ | 0.28 | Piper et al. 2017 | Determines the rate that low SES infected individuals become absent and remove approximately 90% of edges |

References

Carrat F, Vergu E, Ferguson NM, Lemaitre M, Cauchemez S, Leach S, et al. Time lines of infection and disease in human influenza: A review of volunteer challenge studies. Am J Epidemiol 2008;167:775–85. https://doi.org/10.1093/aje/kwm375.

Cohen S, Adler N, Alper CM, Doyle W., Treanor JJ, Turner RB. Objective and Subjective Socioeconomic Status and Susceptibility to the Common Cold. Heal Psychol 2008;27:268–74. https://doi.org/10.1037/0278-6133.27.2.268.

Linn ST, Guralnik JM, Patel KK. Disparities in Influenza Vaccine Coverage in the United States, 2008. J Am Geriatr Soc 2010;58:1333–40. https://doi.org/10.1038/jid.2014.371.

Pepin KM, Riley S, Grenfell BT. Effects of Influenza Antivirals on Individual and Population Immunity Over Many Epidemic Waves. Epidemiol Infect 2013;141:366–76. https://doi.org/10.1016/j.physbeh.2017.03.040.

Piper K, Youk A, James AE, Kumar S. Paid sick days and stay-At-home behavior for influenza. PLoS One 2017;12:1–13. https://doi.org/10.1371/journal.pone.0170698.
