# Supplementary material for: Health inequities in influenza transmission and surveillance
Source: PLoS Comput Biol. 2021 Mar 11;17(3):e1008642. doi: 10.1371/journal.pcbi.1008642 (PMC7951825; doi:10.1371/journal.pcbi.1008642)
Supplement: S5 Table — (DOCX) [file pcbi.1008642.s047.docx]

**Covariate data for Bayesian hierarchical model**

| Role in model | Covariate | Data | Spatial scale | Temporal scale | Source |
| --- | --- | --- | --- | --- | --- |
| Measurement process | Physicians in medical claims database | Medical claims | County | 2002-2008, averaged | Medical claims database |
| Measurement process | Low SES population size | Population size * percent low education | County | 2019 | Population size from US Census Bureau, low education levels from County Health Rankings |
| Susceptibility | Reports of “poor health” | Number of low education people reporting poor health / Number of low education people sampled | County | 2012 | BRFSS |
| Social contact | Household size | Average number of people living in household, reported by low education individuals | County | 2012 | BRFSS |
| Sickness absenteeism | Student chronic sickness absenteeism (absent > 10 days in a school year) | Number of Black students reported absent > 10 days / Number of Black students | County | 2015-2016 school year | US Department of Education |
| Vaccination | Adult influenza vaccination | Number of low education individuals that reported received an influenza vaccination / number of low education individuals | County | 2012 | BRFSS |
| Healthcare access | Insurance coverage | Number of low education individuals that report having health insurance / Number of low education individuals sampled | County | 2012 | BRFSS |
| Healthcare access | Reports having a personal doctor | Number of low education individuals that report having a personal doctor / Number of low education individuals sampled | County | 2012 | BRFSS |
| Healthcare access | Reported avoiding healthcare due to cost | Number of low education individuals that report avoiding healthcare due to cost / Number of low education individuals sampled | County | 2012 | BRFSS |
