# Supplementary material for: Health inequities in influenza transmission and surveillance
Source: PLoS Comput Biol. 2021 Mar 11;17(3):e1008642. doi: 10.1371/journal.pcbi.1008642 (PMC7951825; doi:10.1371/journal.pcbi.1008642)
Supplement: Attachment — Submitted filename: Zipfel_PLOS_comp_bio_response_to_reviewers_11_23.pdf [file pcbi.1008642.s048.pdf]

Health inequities in influenza transmission and surveillance
Zipfel et al.
Response to Reviewers
11/23/2020

Reviewer #1:

Authors in this manuscript characterized the effects of health inequalities on an infectious disease dynamics. They developed a transmission model of influenza and assessed the role of SES-based behavioral and physiological differences on the disease dynamics at the population level.

A network model was developed using ERGM from the POLYMOD social network survey and integrated into the transmission model. The contact network structure accounts for heterogeneity in contact patterns by SES.
Five drivers of disparities in influenza burden that were accounted for in this study were: different social contact patterns, low vaccine uptake, low healthcare utilization, susceptibility, and low sickness absenteeism from school or work.

Further, the authors developed a spatial Bayesian hierarchical model to estimate latent influenza burden in low-SES populations in the United States.

This is a very important topic to focus on, considering the existing health disparities in the country, and this work addresses the association of SES levels with a disproportionate burden of disease and emphasizes the need to focus public health efforts on reducing socioeconomic health disparities.
We thank the reviewer for their positive review of our work.

Looking at the network model goodness of fit figures, such as Figures S8 and S14, the simulated network is not in agreement with the POLYMOD data. Please discuss these results in the text.
We have added the following text to line 373 to highlight that this model produces a reasonable fit for each factor, though each fit individually is not perfect, and it was the best model of those that we compared: “We highlight that we have selected the best fit model, balancing the fit of each incorporated model term. Thus, while each individual attribute may not be an exact fit, this model best captures the main characteristics of each attribute.”

Was the SEIR model calibrated before accounting for the five drivers?
Yes, we assessed the results of the SEIR model prior to incorporating the mechanisms. To demonstrate this, we have added the simulation results with no SES-based

mechanisms occurring on both the regular (control) network, and the SES-heterogeneous network from the ERGM simulation to figure S32.

In the equation for the probability of detection, page 19, please explain what z represents.
We have added an explanation of Z to line 441. The text now reads “We modeled the probability of detection...where α_{0} is the intercept, α_{k} represents \hl{the coefficient estimate for the k measurement process predictor variables, $z_{i, t, k}$} (here, physicians in database and low SES population size), and ν_{c} and ν_{s} are group effects for county and state, respectively.” For consistency and clarification, we also modified the text in line 445 explaining the equation for lambda: “The λ_{i} is modeled by...where β_{0} is the intercept, β_{j} represents coefficient estimates for \hl{the j} low-SES ILI process covariates, \hl{$x_{i,j}$} (here, variables that capture each of the hypothesized mechanisms- susceptibility, social contact differences, absenteeism, vaccination, and healthcare access- in low SES populations), and μ_{c} and μ_{s} represent county-level and state-level group effects, respectively.”

In the spatial model, the model is underestimating the outcome in comparison with the observed outcome. Please address this in the text. It also would be nice to see a time series of modeled vs. observed outcomes rather than scatter plots (Figs 34-37).
In the observed vs. modeled plots, the areas with lower observed low SES ILI have higher modeled values on average, whereas the areas with higher observed low SES ILI have lower modeled values on average. This is the result of the measurement process. High observed low SES ILI counts are likely to occur in counties that have better surveillance and thus higher measurement in the model. Thus, the modeled results for these high observation counties are scaled down, while those with less observation are scaled up, to counteract poor measurement. We added text in line 454 to explain this point: “We highlight that areas with high observed low SES ILI are underestimated by the model, due to measurement being efficient in these areas \ref{obs_vs_mod}. This indicates that our estimates of low SES ILI in these areas may be conservative.” This model is not a temporal model, thus a time series of modeled vs. observed outcomes is not possible.

Page 20, lines 423-424, the covariate values were assumed to be constant over time from 2002-2008. Is it possible to develop the model for each year separately? Or develop a model with low, medium, and high values of covariates over the 2002-2008 time period? How would this change the results?
We agree that further development of such a model to examine temporal differences would be very useful. However, we are currently limited by the availability of temporally

varying covariate data that is specific to low SES populations. In the model, all of the data is pertinent only to low SES populations (i.e. we include only the vaccination uptake of low SES individuals, etc.). The data from BRFSS (all covariates besides sickness absenteeism) is only reported at the individual level, and thus able to be parsed by SES group, in 2012. The sickness absenteeism data is only reported for a limited time period as well. Additionally, influenza dynamics are highly variable from season to season for a variety of ecological and virological reasons. We believe attempting to attribute these annual fluctuations to socioeconomic factors, which tend to be relatively stable from year-to-year, would be an overinterpretation of our findings. We clarify this point in line 290: “While there may be variation in the dynamics of ILI among low SES populations over time, time-varying data on our covariates is currently unavailable and we do expect socioeconomic factors and health behaviors to remain relatively consistent across seasons.”

Page 21, lines 460-461: the missing covariate values were imputed. Could you please provide the percentage of data that was missing?
We calculated this percentage and added it to the text in line 509: “Approximately 32\% of the counties in the included states had a missing covariate data value, and thus were imputed.”

Reviewer #2:

This is an ambitious paper on a very important topic. It uses a mix of dynamical modeling and ecological statistical analysis to highlight the multiplicative effects of low SES on both flu burden and our persistent inability to observe the burden accurately. Especially at this moment where the COVID19 epidemic has laid bare the costs of systemic health inequity, this work using epi modeling and historical knowledge from flu adds useful evidence to advocate for more representative disease surveillance and more focused disease control.

The use of "intersectionality" is appropriate, and I appreciate bringing the language of social justice into model-heavy epidemiology, where it both belongs and was (to me) jarring at first. But it also motivates a question that readers will ask throughout -- are any inferences in this work likely to causal or even identifiable? Systemic inequity manifests in how many plausibly causal factors collide in the same population, and how non-causal factors often explain the most variance.

The dynamical-model-based exploration of how social factors coincide to drive transmission builds a clear and plausible case for how the factors pile together in the

same direction, with reasonable numerical effect sizes. This speaks nicely to the compounding effects that can be quantified while individual causal factors cannot be described independently (as in the sense of a regression coefficent, all else held equal). This is the strongest part of the paper.

My main criticisms are in relation to that, in relationship to the ecological analysis that veers into causal over-interpretation in some places I flag below. Where noted, I think the storytelling subtracts more than it adds and so I encourage the authors to more carefully describe what can be learned from their analysis and what remains unidentifiable. To their point about intersectionality and the need for more effort to address the issues raised, not being able to answer the questions today because of fundamental statistical issues is one manifestation of the inequity they are shining the light on.

The open sharing in github is very welcome. I continue to be happy to see teams supporting this mode of science communication. I haven't vetted the code carefully, but I skimmed through some key functions and feel comfortable saying it is readable with a reasonable flow -- good for reproducibility and likely can be understood by a motivated reader. I appreciate the verbose variable names in the SEIR code in particular, I recognize the irritation of getting INLA outputs into a useable form, and must however acknowledge that the network code is noticeably less easy to make sense of.

I apologize that the review contains some comments I may be able to answer for myself given time -- this is a technically sophisticated effort that requires close attention to evaluate. COVID never sleeps but I must...

Overall I think this is an important paper that speaks to very pressing issues, and the methods are appropriate when interpreted judiciously. I look forward to seeing it in print after revision.
We appreciate the careful read and positive feedback.

Major comments
37-39: It is correct to point out that all the factors are synergistic, but I'm not sure it's right to say that addressing one may alleviate others. Because one can't identify the relative contributions of each cause, it is possible to target the least important one and thus have very little benefit for the effort. Arguably this is the norm when targeting inequity in the US. I suggest going with a less causal statement.
We have omitted this statement.

When starting the results discussion around "low SES ILI", please be more clear about the meaning of terms. Lines 188 & 194 for example drop parenthetical definitions that are hard to keep track of in total. I take away that you define SES in terms of education only, and then look at ratios of ILI per hospital visit vs SES, but I'm not sure on first reading. This would also help (at least me) with my confusion about how the regression works (see my comment about line 412). I don't fully understand the outcome variable (it should be a count per the equation in line 393 but it appears to be a rate ILI/visits/1000??). The implementation in the code makes reference to an offset (https://github.com/bansallab/fluSES/blob/2624f6ade4230f94f1485a7590a69a10a5469a1a/Statistical%20Model/best_low_ses_county_inla_model_7_1.R#L187) so I assume the ratio is being done in a sensible way, but I don't have time to download and test myself nor should that be necessary.

We have made the sections in which low SES ILI is introduced in the context of the spatial model more clear. First, where the relationship between the ILI data and county low SES populations is introduced, the text in line 201 has been modified to: "However, when we compare county ILI burden with the proportion of the county's population of low SES, we find a negative relationship, indicating lower levels of influenza in counties with larger low SES populations". Then, we more clearly define the calculation and reasoning behind the outcome variable in line 208: "Here, we define low SES ILI as an incidence ratio, where low SES ILI cases are normalized by the number of 1000 visits, to account for spatio-temporal variation in database coverage and healthcare-seeking. Thus, the model outcome data is the rate of ILI healthcare visits per 1000 healthcare visits within each county. " Because we have included the coverage/healthcare-seeking in the response, an offset is not necessary. We have removed that line from the Github code to prevent misunderstanding.

Figure suggestion: can you show the low SES ILI incidence ratio vs the low SES variable? Or some other scatter plot that shows how visits fall off with SES and percent ILI rises. Something to emphasize the competing effects and clearly define the derived variable that is the outcome in the regression. This could strengthen the narrative about data inequity itself masking burden.

We have added figure S39, which demonstrates how A) ILI cases decrease with increasing low SES population, B) total visits decrease with increasing low SES population, and C) how the low SES ILI incidence ratio related to the low SES population, both in the observed data and in the modeled rate. This leads to omission of 2 other supplemental figures, which were made redundant by this figure. We reference this figure S39 in lines 203, 205, and 213.

230-241: Here is where the causal storytelling I was worried about up front takes place! For example, household size is a strong predictor of transmission risk for COVID and also should be from a network perspective. So for household size to be be negatively associated with ILI risk, either the hypothesized social determinants are either stronger than the physical ones, or it is confounded with some unknown covariate (like how clusters with similar SES but different ethnic or religious backgrounds may have different family structures, and this covaries with geography too). Similarly, for flu vax, it's not unusual to find flu vax to be positively correlated with flu incidence. This can easily reflect general health-seeking behavior and not just vax-seeking due to risk, and thus there could be selection effects that go beyond what is scaled for with the total visits. The overall positive association with healthcare utilization factors points in this direction. My point is this paper has a strong message about colinear synergistic effects clearly aligning to enhance burden on low SES people, and low SES minimizes our system's ability to see that risk in data. That message is well told and in my opinion it is harmed by further adding on unsupported and incomplete causal scenarios.

We have edited the text accordingly:

Line 125: “These findings also indicate potential SES-based factors associated with disproportionate burden at the population level, which could guide future public health efforts to reduce socioeconomic health disparities.”

Line 224: Susceptibility and sickness absenteeism differences may be associated with ILI in low SES populations”

We have removed the interpretation of non-significant coefficients and tempered the causal language in this section, starting on line 226: “Fig \ref{fig4} shows the coefficient estimates and credible intervals resulting from the Bayesian spatial hierarchical model. Levels of poor health among low SES individuals, as a measure of susceptibility to infection, are positively related with low SES ILI incidence. Thus, areas with higher reports of poor health among low SES individuals are associated with higher burden of ILI among low SES populations. Also, access to sickness absenteeism among low SES individuals, represented by the number of low SES students that are absent for more than 10 days in a school year, is negatively related to low SES ILI incidence rates. Thus, areas where more low SES students are able to be absent are associated with lower rates of low SES ILI.”

We have also added a paragraph to the discussion that addresses the reviewer’s concerns, starting on line 298: “A main limitation of the spatial inferential model is the identifiability of separate effects. Here, we have identified possible associations in our model, but this is only the start to disentangling the factors that contribute to health inequities. The lack of significant association with the other incorporated covariates does not indicate that these are not important to inequities in influenza transmission in low SES populations. These impacts may be obscured by several issues. The covariate data may be impacted by its own biases, insufficient sample sizes, and other limitations.

When ubiquitous systemic inequities go unaccounted for in data collection and processing, the signal of low SES individuals may be obscured. We aimed to counteract this by only using covariate data specific to low SES populations, but this was parsed out from data collected for the whole population that included demographic data, identifying potentially lower SES individuals. Next, there may be other factors relating to increased influenza transmission that may not be identifiable when focused on mechanistic explanations, and the model may not be able to parse synergistic factors. Our network epidemic model demonstrates that multiple factors of inequity can compound one another non-linearly, and statistically identifying individual effects remains a challenge due to lack of data and statistical limitations. Further attention to systemic inequities in health and epidemiology will be necessary to move this problem forward."

254-267: great paragraph.
Thank you!

412: I'm confused about how the response can be normalized in a regression where the response variable is supposed to be a count. I'm not sure where my misunderstanding is arising, so please edit for clarity. Is it just that the variable is defined such that it's always positive and INLA handles the analytic continuation to non-integer counts gracefully?
We address this comment above with a clearer explanation in the results section. We also added an example to line 466 in the methods so the calculation will not be misunderstood by readers and the logic is much clearer: "As an example, a hypothetical county composed of 40\% low SES individuals has 500 total ILI visits out of 8,000 total healthcare visits. Figure \ref{fig2}B shows that a county with 40\% low SES in the population is expected to have about 55\% of ILI cases in low SES individuals. Thus, we estimate this county's low SES ILI cases as 275 ILI cases, which we normalize per 1000 total visits, resulting in a rounded ILI incidence ratio of 34."

Minor copy-editing required throughout. Like line 12, period-space before "Here".
Thank you for drawing our attention to this. We have corrected this, and conducted additional proofreading.

Figure 2B needs a color legend on the figure itself.
We have added a legend to figure 2B.

416 and 423: "Inla does not allow..." in N-mixture models. Unless I'm misunderstanding what is meant by 'measurement covariates", this is not a general statement for all INLA models.

Indeed, N-mixture models in INLA cannot handle temporally varying measurement covariates as discussed by the package authors in “Estimating Animal Abundance with N-Mixture Models Using the R-INLA Package for R”. However, we have removed that sentence from the methods lest it add any confusion.

I did not evaluate the dynamical model code, but the method as described makes sense and the many supplemental figures document convincingly (to me) that the model is likely behaving as intended.
Thank you.

Reviewer #3:

The manuscript analyzes the impact of socio-economic disparities in the spread of seasonal influenza in US. Authors combine mechanistic modeling of influenza spread on a contact network with statistical analysis of influenza incidence records across space. This allows them to quantify the relative role of different mechanisms determining increased spreading risk for low socioeconomic status (SES) individuals and to map health disparities in space.

The topic is an important and timely one. The manuscript presents an extensive analysis that combines different data sources and methodologies. I believe that the work has the potential to provide a nice contribution to PLOS Computational Biology. However, several improvements are needed especially in the presentation of the work that is at this stage unclear in many parts. Also, some hypotheses should be discussed more in depth, and alternative parameters should be explored in a sensitivity analysis. I detail in the following the major points to be addressed
We thank the reviewer for their positive feedback.

1) The work is extensive and methods used are rich and complex. I believe that the methodological part should be put in prominence and should be presented before the results. Also, I could not follow the presentation of the results without reading the methods first.
The PLOS Computational Biology submission guidelines indicate that papers should be formatted with the Materials and Methods section following the results. In accordance with these guidelines, we have kept the existing format. We have edited the first paragraph of the results section to more clearly summarize the methods, and added additional explanation of steps taken throughout the results section.

2) More in general I believe that the paper should be restructured. Some parts of the methods are discussed in Results (e.g. end of page 10 when model validation is

discussed). The Results section contains also some parts of the model discussion and limitations that should go on the Discussion (e.g. end of page 12, regarding the discussion of the vaccination result).

As described above, we thought it would be most in keeping with submission guidelines to not restructure the paper. We believe this necessitates brief methods summaries in the results section to guide the reader throughout the presentation of the results. We have moved the identified lines to the discussion, starting on line 268:

“Low SES absenteeism is here measured by student absenteeism, which may not be a perfect measure of sickness absenteeism or paid sick leave access. However, other fine-scale data was lacking, and a student’s ability to be absent is related to a parent’s ability to be home to care for the child, and differences in access to paid sick leave by SES have been related to student sickness absenteeism levels due to influenza [38, 39, 40, 41]. To validate our findings, we compared the trends in our model estimates to previous estimates of influenza incidence ratios, stratified by poverty level. This is not a direct comparison, as previous studies present the incidence ratios for the entire population, not just for low SES individuals within those populations. Our results show more consistently high incidence ratios compared to the larger increases between poverty rates in prior studies. We attribute this to the incorporation of the measurement process into our models, which accounts for undersurveillance of low SES infection, whereas healthcare access and healthcare seeking differences may have missed low SES cases in prior studies. Ideally, data on respiratory infection of low SES individuals would be available at a fine spatial scale to more directly assess the validity of our models, but the lack of such a dataset highlights the need for future surveillance and data collection that focuses attention on lower SES populations.”

3) Many details are missing from the methods:

In addition to addressing the gaps highlighted, we have also added additional details to the methods, in an effort to clarify details and logic.

a. Authors mention that the ERGM model is fitted to POLYMOD data. What observable is fitted?

We clarify the model structure in line 368: “The best model observed data was the egos and their alter contacts. Model terms included edges, node attributes for gender, age, school/work, and education, and homophily for age, home, school/work, and education.”

b. In the description of the SES-based epidemiological model author write that delta and delta_low are respectively vaccination coverage in high and low SES individuals, however in TableS4 is written “general vaccination rate” for delta. This “general is confusing”, it points to an average quantity over the whole population. Similarly, for beta, gamma and roh it is not clear if these quantities are averages over the whole population or only high-income individuals

We have clarified this in several places. First, we change all instances of a parameter to either $parameter_high$ or $parameter_low$. Then in the methods, we added line 413: “The high parameters were applied to medium and high education nodes, and the low parameters were applied to low education nodes, as defined in the ERGM model above.”

c. Table 4S should be presented in the main text.
We thank the reviewer for the suggestion, however we believe that including the Table in the main text would unnecessarily interrupt the flow of presentation of methods and results. Presenting the table with model parameters and associated values in the SI is a rather standard practice.

d. How are the spreading simulations performed? More precisely: how are initial conditions defined? How long is the epidemic period (single season/multiple seasons)? Are people staying at home when infectious and how is this modelled in practice?
We have added text to the Methods section to more clearly define these issues in line 416: “We assume a naive population of entirely susceptible individuals, and each simulation represented one influenza season, continuing until there were no new exposed individuals. We note that the only isolation of infected individuals that occurs is when absenteeism is incorporated into the simulation, as described above.”
We have also tested multiple influenza seasons to build immunity in the population, and showed that findings do not change. This is explained in detail in response to comment 4b of the reviewer (here below).

e. I had some difficulty in following the description of the Bayesian hierarchical model. Process predictor variables and covariates should be introduced immediately after the equations (at least briefly). I felt like plenty of details are given without a clear introduction of the overall methodology. Also, the way in which the two parts (network model and statistical analysis) are combined should be better presented.
We have clarified this section in the following ways.
First, we introduce the covariates for the measurement and process models immediately after the equations:
Line 440: “We modeled the probability of detection $p_{i,t}$ as: ...where α_{0} is the intercept, α_{k} represents \hl{the coefficient estimate for the k measurement process predictor variables, $z_{i, t, k}$} \hl{(here, physicians in database and low SES population size)}, and ν_{c} and ν_{s} are group effects for county and state, respectively. “
Line 444: “The λ_{i} is modeled by: ...where β_{0} is the intercept, β_{j} represents coefficient estimates for \hl{the j} low-SES ILI process covariates, \hl{$x_{i,j}$} \hl{(here, variables that capture each of the hypothesized

mechanisms- susceptibility, social contact differences, absenteeism, vaccination, and healthcare access- in low SES populations)}, and μ_{c} and μ_{s} represent county-level and state-level group effects, respectively. “

Second, we provide more of an introduction to the spatial model section:

Line 437: “This is an N-mixture model, which accounts for imperfect detection of low SES ILI cases through a measurement process, as well as borrowing information from county-level factors associated with influenza in low SES populations. The goals of this model are to estimate cases of ILI in low SES populations in counties across the United States accounting for measurement processes and data on low SES behavioral and physiological differences, and to identify the relationship between the hypothesized drivers of inequities and low SES ILI at the population level.”

Lastly, we added an example to the Response Data section to more clearly explain how and why we incorporate the network model findings with the spatial model.

Line 466: “As an example, a hypothetical county composed of 40\% low SES individuals has 500 total ILI visits out of 8,000 total healthcare visits. Figure \ref{fig2}B shows that a county with 40\% low SES in the population is expected to have about 55\% of ILI cases in low SES individuals. Thus, we estimate this county's low SES ILI cases as 275 ILI cases, which we normalize per 1000 total visits, resulting in a rounded ILI incidence ratio of 34.”

4) Some assumptions should be discussed more in detail. Here are some assumptions/choice that I believe could be better motivated or would be benefit from sensitivity analysis.

a. Some sensitivity analysis on the parameters reported in Table S4 should be conducted. In particular I am referring to the ones related to the differences in transmission between high and low SES individuals.

We identified the parameter values from relevant literature, but we have also conducted sensitivity analysis by varying the lower SES parameters in their full range of values, and have added the results as Figures S35-S38 in the supplement. Results are robust against changes of these values. These results are included in the text in line 388.

b. Sensitivity should be conducted also on modelling assumptions. In particular, if I correctly understood authors assume that the whole population is naïve to the virus. In the modelling framework used by the authors, pre-existing immunity can be absorbed on the transmissibility parameter beta. However, some level of heterogeneity may in principle exist on the level of immunity among different SES groups. Authors should discuss this point, and test alternative scenarios.

We have tested this scenario by simulating 5 subsequent seasons of influenza with polarized partial immunity amid the SES-based behavioral and physiological differences. We find that the proportion of the infected population remain

disproportionately composed of low SES individuals. We added 2 supplement figures (S33 and S34) demonstrating these results. We have added this step to the methods (starting in line 426) and results (starting in line 189) as well.

c. Authors state “we resampled additional low education egos from the low education sample in the POLYMOD dataset”. This is not completely clear to me. Did authors test different proportion of low SES individuals in the population? What is the reasoning behind that?

Yes, we wanted to evaluate how the SES composition of the population might impact the epidemic model results. For example, would populations with larger low SES populations experience the impacts of SES-based differences non-linearly? This is important to inform on the expected impact of low SES individuals across territories with different SES profiles. We have added a clarifying line 362: “These networks allow us to examine how epidemic dynamics might differ in populations with different proportions of low SES individuals in the population (capturing e.g. the SES variability observed in the United States).”
